# The Construction and Analysis of Immune Infiltration and Competing Endogenous RNA Network in Acute Ischemic Stroke

**DOI:** 10.3389/fnagi.2022.806200

**Published:** 2022-05-17

**Authors:** ZhaoLei Ma, Chun-Feng Liu, Li Zhang, Ning Xiang, Yifan Zhang, Lan Chu

**Affiliations:** ^1^Department of Neurology, The Affiliated Hospital of Guizhou Medical University, Guiyang, China; ^2^Department of Neurology, Clinical Research Center of Neurological Disease, The Second Affiliated Hospital of Soochow University, Suzhou, China; ^3^Institute of Neuroscience, Soochow University, Suzhou, China; ^4^Department of Geriatrics, The Affiliated Hospital of Guizhou Medical University, Guiyang, China

**Keywords:** ischemic stroke, ceRNA, immune infiltration, inflammation, neutrophils

## Abstract

Acute ischemic stroke (AIS) is a common neurological disease that seriously endangers both the physical and mental health of human. After AIS, activated immune cells are recruited to the stroke site, where inflammatory mediators are released locally, and severe immune inflammatory reactions occur within a short time, which affects the progress and prognosis of IS. Circular RNA (circRNA) is a type of non-coding RNA (ncRNA) with a closed-loop structure and high stability. Studies have found that circRNA can affect the course of IS. However, there is no report on ceRNA’s pathogenesis in AIS that is mediated by circRNA. In this study, the CIBERSORT algorithm was used to analyze the distribution of immune cells in patients with AIS. mRNA dataset was downloaded from the GEO database, and the weighted gene co-expression network analysis (WGCNA) method was used to construct weighted gene co-expression to determine 668 target genes, using GO, KEGG enrichment analysis, construction of protein-protein interaction (PPI) network analysis, and molecular complex detection (MCODE) plug-in analysis. The results showed that the biological function of the target gene was in line with the activation and immune regulation of neutrophils; signal pathways were mostly enriched in immune inflammation-related pathways. A Venn diagram was used to obtain 52 intersection genes between target genes and disease genes. By analyzing the correlation between the intersection genes and immune cells, we found that the top 5 hub genes were TOM1, STAT3, RAB3D, MDM2, and FOS, which were all significantly positively correlated with neutrophils and significantly negatively correlated with eosinophils. A total of 52 intersection genes and the related circRNA and miRNA were used as input for Cytoscape software to construct a circRNA-mediated ceRNA competition endogenous network, where a total of 18 circRNAs were found. Further analysis of the correlation between circRNA and immune cells found that 4 circRNAs are positively correlated with neutrophils. Therefore, we speculate that there may be a regulatory relationship between circRNA-mediated ceRNA and the immune mechanism in AIS. This study has important guiding significance for the progress, outcome of AIS, and the development of new medicine.

## Introduction

Stroke is one of the major diseases that cause death and disability worldwide. Statistically, about 15 million people worldwide suffer from stroke every year (Li et al., 2018). With the rapid growth of the aging population, the incidence and mortality of stroke increase progressively each year. Notably, acute ischemic stroke (AIS) caused by cerebral artery thrombosis or embolism accounts for about 80–85% of stroke cases (Jian et al., 2019). So far, the most effective treatments for AIS are intravenous thrombolysis within the “time window” (Powers et al., 2018) and intravascular interventional therapy (Goyal et al., 2015). However, only a few patients have benefited from these treatments, and there is a high level of risks associated with these treatment methods. Therefore, the identification of the molecular mechanism of AIS pathogenesis, the screening of effective and reliable biological markers for early screening and AIS diagnosis, and timely and accurate intervention for patients with AIS are essential to reduce the incidence and the poor prognosis.

Recent studies have found that immune cell-mediated inflammatory response plays an important role in the pathogenesis of AIS. Following the stroke, the body’s innate immune system and adaptive immune system are activated. Inflammatory responses are also active during the entire pathophysiological process of AIS, which forms one of the important pathophysiological processes after AIS (Fu et al., 2015). The possible mechanism is through the damage-associated molecular patterns (DAMPs) released by damaged neurons after stroke, which triggers local immune responses that lead to glial cell activation and peripheral leukocytes recruitment to the damaged area, and the secretion of a variety of pro-inflammatory cytokines, chemokines, and matrix metalloproteinases (MMPs), which cause the damage to the blood-brain barrier, cerebral edema, hemorrhagic transformation, and neuronal necrosis (Rajkovic et al., 2018; Yang et al., 2019).

Non-coding RNAs (ncRNAs) include microRNAs (miRNAs), long non-coding RNAs (lncRNAs), and circular RNAs (circRNAs), which can regulate protein coding through a variety of mechanisms. This has an important role in the pathophysiology and recovery of angiogenesis after AIS (Ding et al., 2020; Gan et al., 2021; Li J. et al., 2021). Competing endogenous RNA (ceRNA) has miRNA binding sites and can compete with mRNA to bind miRNA to construct a circRNA-miRNA-mRNA regulatory network, thereby inhibiting miRNA regulation of target genes. The mechanism of ceRNA is complicated. For example, circRNA can bind miRNA for ceRNA, thereby preventing miRNA from inhibiting target genes (Salmena et al., 2011). Studies have shown that circTLK1 as a ceRNA can inhibit the activity of miR-335-3p and cause neuronal damage. circTLK1 knockout can significantly reduce the cerebral infarct volume of MCAO mice, reduce neuronal damage, and improve subsequent long-term neurological deficits (Wu et al., 2019).

The role of lncRNA-mediated ceRNA in the immune pathogenesis of AIS has been reported previously (Li S. et al., 2021). However, there is no report on the immune mechanism of circRNA-mediated ceRNA in AIS. This study aims to explore the distribution characteristics of immune cells in patients with AIS, to explore the role of circRNA-mediated ceRNA regulatory network in the immune mechanism of AIS, and to provide a new path for the pathogenesis and treatment of IS research.

## Materials and Methods

### Distribution Characteristics of Immune Cells and Their Relationships in Patients With Acute Ischemic Stroke

CIBERSORT is currently the most used immune cell infiltration estimation and analysis method (Newman et al., 2015). CIBERSORT^1^ algorithm was used to analyze RNA-seq data from different patient subgroups and to infer the composition ratio of 22 types of immune infiltrating cells. The default matrix was set to 1,000 permutations, and samples with *p* < 0.05 showed significant differences. The *p*-value of CIBERSORT can reflect the statistical significance of the deconvolution results of all cell subsets, which was used to filter out the deconvolution results with less significant fitting accuracy. To evaluate the difference of immune cell infiltration between the stroke group and the control group, “pheatmap” package was used to draw the heatmaps of immune cell infiltration to explore the distribution of immune cells between high- and low-risk groups. “corrplot” software package was used to analyze the interactions between immune cells and the impact of these interactions. “vioplot” software package was used to plot the relative content of immune cells.

### Preprocessing and Analysis of Microarray Dataset

In this study, the original circRNA, miRNA, and mRNA gene expression matrix datasets were downloaded from the gene expression database, i.e., GEO.^2^ circRNA dataset (GSE133768) (Yang et al., 2020) included 3 disease groups and 3 healthy control groups. The sequencing platform used was GPL21825[074301] Arraystar Human circRNA microarray V2 (Agilent Technologies, Rockville, MD, United States). The mRNA dataset (GSE58294) is composed of 92 mRNA sequencing data (Stamova et al., 2014), including 69 disease groups and 23 healthy control groups. The expression profile sequencing platform used was GPL570 [HG-U133_Plus_2] Affymetrix Human Genome U133 Plus 2.0 Array (Affymetrix, Santa Clara, CA, United States). R limma package (Smyth, 2005) was used to detect the differential expression of circRNAs and mRNA between the disease group and the healthy control group, respectively. ggplot2 package (Davis and Meltzer, 2007) and Complexheatmap package were used (Gu et al., 2016) for the visualization of heatmaps and volcano maps to show the significant expression of DEcircRNAs and DEmRNAs (|logFC| > 1 and *p* < 0.05 are considered as significant differences).

### Gene Ontology and KEGG Pathways Analysis of Differentially Expressed Genes

The DAVID database^3^ (Rameshwari and Prasad, 2012) that integrates biological data and analysis tool and performs functional enrichment analysis based on the DAVID database, including gene ontology (GO) annotations and gene encyclopedia (KEGG) enrichment analysis, was used in this study. GO analysis divides the gene function into three categories: biological process (BP), cell component (CC), and molecular function (MF). Based on the above three aspects, we can get gene annotation information (Harris et al., 2004). KEGG enrichment analysis can help us understand the signaling pathways in which the differentially expressed genes (DEGs) have been involved.

### Protein-Protein Interaction Network Building for Target Genes and Biological Process Analysis

The study of mutual network between proteins helps to identify the core regulatory genes. Currently, the Search Tool for the Retrieval of Interaction Genes (STRING) database (von Mering et al., 2003)^4^ is the highest species coverage and the largest interaction. A protein-protein interaction (PPI) network was constructed based on STRING. In this study, the PPI network of DEGs was constructed based on the minimum interaction (>0.4). PPI network was uploaded to the Cytoscape software (version 3.7.2) for visualization (Shannon et al., 2003).

### Module Analysis by Using Molecular Complex Detection Plug-In

Molecular complex detection (MCODE) plug-in Cytoscape software was used to identify closely related modules in the PPI network: cutoff value was set at 2, and the node score cutoff value was 0.2. The advantage of the algorithm of this module is that it will not be affected by a high proportion of false positives caused by high-throughput technology. In addition, GO and KEGG analysis of genes in the PPI network module were performed through the “clusterprofiler” package in the R software. The value of *p* < 0.05 indicated a significant difference.

### Determine the Final Intersection Genes by Venn Diagram

The final intersection genes through the Venn diagram have undergone GO and KEGG analysis using the Metascape database.

### Correlation Analysis of Hub Genes and Immune Cells in Patients With Acute Ischemic Stroke

The NetworkAnalyzer in Cytoscape (version 3.7.1) was used to analyze the topological parameters in the network, select the best target according to the degree and the top 5 as hub genes, and analyze the correlation between the hub genes and the immune cells.

### Construct Receiver Operating Characteristic Diagnostic Curve to Verify the Diagnostic Value of Hub Gene

The pROC package in the R language was used to perform receiver operating characteristic (ROC) on the selected specific genes. The area under the curve (AUC) of the core gene was compared to verify the core gene’s ability to predict disease sensitivity and specificity; finally, ggplot2 package was used for ROC curve visualization.

### Construction of CircRNA-Mediated Competing Endogenous RNA Regulatory Network

CircInteractome data^5^ is a commonly used database for querying the correlation between circRNA and miRNA. Target miRNA prediction for AIS was based on the top 10 most significant positive and negative correlations circRNAs with a statistical difference. Prediction of the interactions between miRNA and mRNA was based on miRDB, miRTarBase, and TargetScan. Combining circRNA-miRNA interactions and visualization of mRNA-miRNA interaction through Cytoscape, a circRNA-miRNA-mRNA network was established. Analysis of the correlation between circRNA and immune cells.

The Shapiro–Wilk normality test was used to test the normality of the data for the correlation analysis. For data with normal distribution, Pearson analysis was used. For data without normal distribution, Spearman’s correlation analysis was used. Ggplot2 (3.3.3 version) was used for visualization. −1 ≤ *r* ≤ 1, where a positive *r*-value indicates a positive correlation and a negative *r*-value indicates a negative correlation.

### Statistical Analysis

All statistical analysis was performed in the R language (version 3.6). All statistical tests were two-sided, and *p* < 0.05 was considered statistically significant.

## Results

### Distribution of Immune Infiltration Levels in Patients With Acute Ischemic Stroke

**FIGURE 1:**
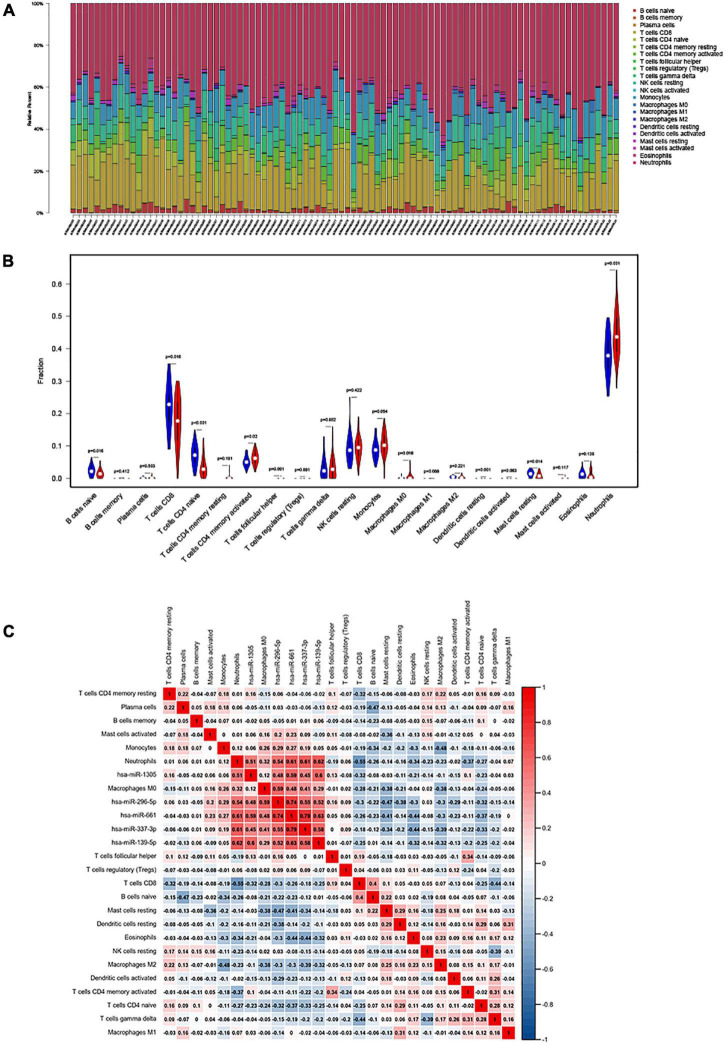
**(A)** The fraction of 22 subsets of immune cells in acute ischemic stroke (AIS), of which the highest proportion is neutrophils; *x*-axis: each GEO sample; *y*-axis: percentage of each kind of immune cells. **(B)** Violin plot showing differences in immune infiltration between normal and diseased groups. Blue showed the normal group and red showed the disease group. Resting dendritic cells, neutrophils, naive CD4^+^ naive T cells, and T follicular helper cell revealed the largest differences between the two groups (*p* < 0.001). **(C)** The co-expression heatmap illustrated significant co-expression patterns about key members in the ceRNA network and key members in the immune cells.

We used CIBERSORT to estimate the distribution ratio of 22 immune cell subsets in AIS, of which neutrophils accounted for the highest proportion (Figure 1A). The results of violin graph showed that the immune infiltration levels of naive B cells, CD8^+^ T cells, naive CD4^+^ T cells, and resting mast cells were downregulated in patients with AIS, while the immune infiltration levels of activated memory CD4^+^ T cells, macrophages M0, and neutrophils were upregulated. Resting dendritic cells, neutrophils, naive CD4^+^ T cells, and T follicular helper cells were significantly different in patients with AIS and normal controls (Figure 1B).

### Interaction Between Immune Cells in Patients With Acute Ischemic Stroke

The Pearson’s correlation coefficient was used to analyze the correlation between immune cells (Figure 1C). Among them, CD8^+^ T cells were strongly positively correlated with naive B cells (0.43), and resting NK cells were moderately positively correlated with memory B cells (0.32). Memory CD4^+^ T cells are moderately positively correlated with gamma-delta T cells (0.25), naive B cells are moderately positively correlated with resting dendritic cells (0.26), CD8^+^ T cells are strongly negatively correlated with neutrophils (−0.6), CD8^+^ T cells and gamma-delta T cells were moderately negatively correlated (−0.43), and macrophages M2 and monocytes were moderately negatively correlated (−0.42).

### Download the Circular RNA and mRNA Datasets From the GEO Database to Screen Differentially Expressed Genes

**FIGURE 2:**
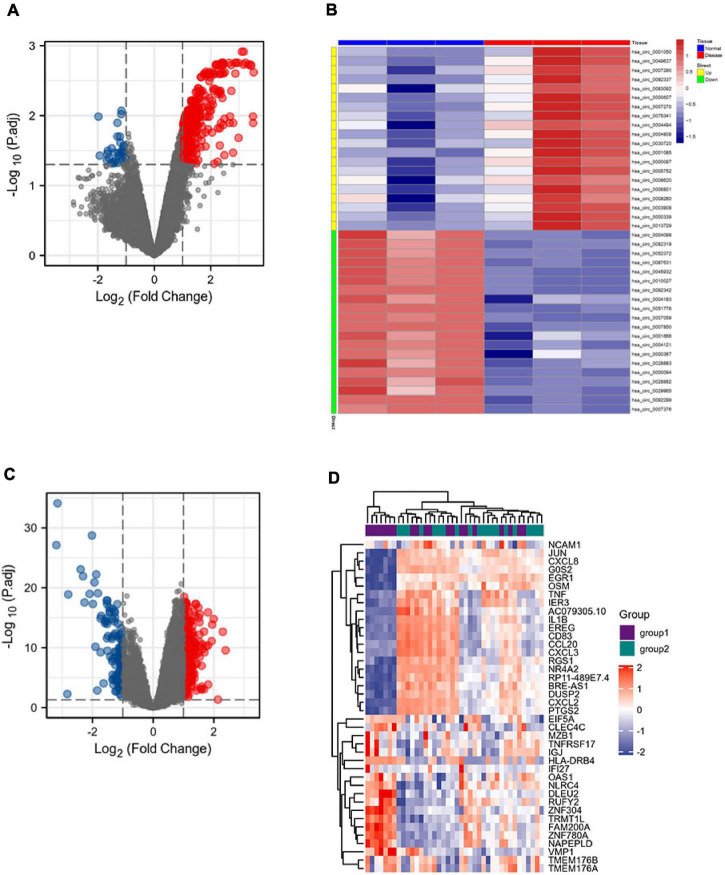
DEGcircRNAs gene expression shown in panel **(A)** volcano map and **(B)** heatmap, including 213 upregulated differential genes and 346 downregulated differential genes. DEmRNAs gene expression shown in panel **(C)** volcano map and **(D)** heatmap, including 198 upregulated differential genes and 121 downregulated differential genes.

The GSE133768 dataset was downloaded from the GEO database. A total of 559 differential circRNAs (213 upregulated and 346 downregulated) were screened out based on the set threshold (*p* < 0.05 and |Log2FC| > 1). The mRNA expression data come from 92 mRNA sequencing data of the GSE58294 dataset. Volcano maps of DEGcircRNAs and DEGmRNAs were shown in Figure 2.

### Use Weighted Gene Co-expression Network Analysis for mRNA to Construct Weighted Gene Co-expression and Find the Gene Modules That Are Most Relevant to the Disease

**FIGURE 3:**
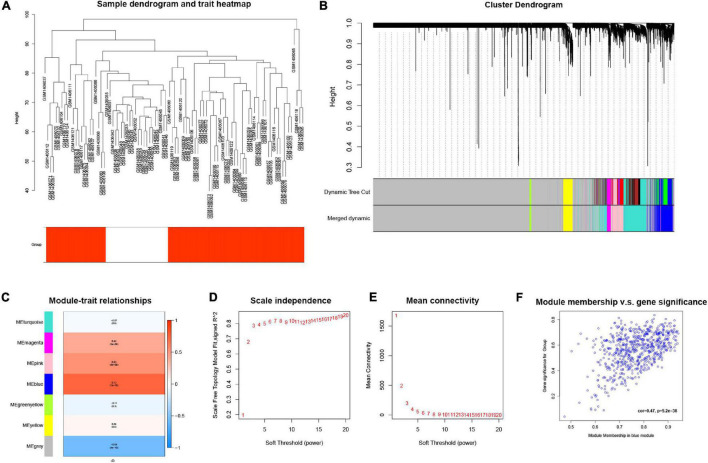
**(A)** Cluster tree drawn using the weighted gene co-expression network analysis method. Each branch represents a gene; *y*-axis represents the cluster distance. **(B)** Cluster dendrogram with soft threshold and 7 merged modules: 668 genes in blue module, 60 genes in green-yellow module, 7,130 genes in gray module, 153 genes in magenta module, 550 genes in pink module, 1,116 genes in turquoise module, and 323 genes in yellow module. **(C)** Module-trait relationships, where blue module has the highest correlation with diseases (cor = 0.73, *p* = 1e^–16^). **(D)** Scale independence; *x*-axis represents the value of power and *y*-axis represents R. **(E)** Mean connectivity; *x*-axis represents the value of power and *y*-axis represents the average connected number of all nodes. **(F)** Correlation between genes in blue module and corresponding traits (cor = 0.47, *p* = 5.2e^–38^).

The GSE58294 dataset, composed of a total of 92 mRNA sequencing data, was downloaded from the GEO database, and an mRNA expression matrix was obtained. The weighted gene co-expression network analysis (WGCNA) method was then used. In general, a weighted gene co-expression network (Figure 3A) was constructed; cooperatively expressed gene modules and the correlation among gene expression networks, phenotypes, and the core genes in the co-expression network were explored; and the WGCNA-R package was used to screen out the top 10,000 genes in variance for further analysis. The results showed that the blue gene modules were most relevant to the disease (Figures 3B–F).

### Perform Gene Ontology and KEGG Enrichment Analysis on the 668 Genes of the Blue Module to View Their Biological Functions

**FIGURE 4:**
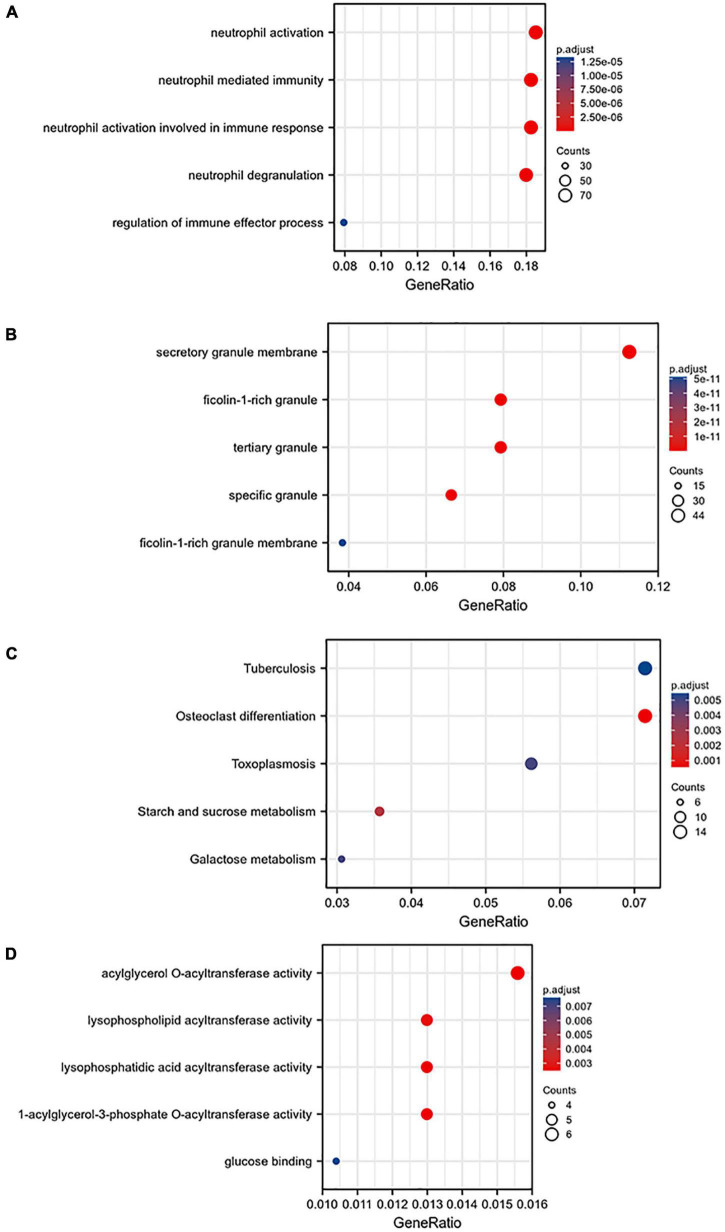
Gene ontology (GO) analysis of the blue module: **(A)** BP, **(B)** CC, **(C)** MF, and **(D)** KEGG pathway enrichment analysis of the blue module.

A total of 668 genes in the blue module were analyzed for GO and KEGG enrichment analysis. Under the conditions of adjusted *p*-value < 0.1 and *q*-value < 0.2, there were 377 BP, 40 CC, 11 MF, and 31 KEGG. The specific functions are shown in Table 1 and Figure 4.

**TABLE 1:** Blue module gene biological function and pathway enrichment analysis.

GO/KEGG	Function/pathway
BB	Neutrophil activation, neutrophil mediated immunity, neutrophil activation involved in immune response, neutrophil degranulation, regulation of immune effector process;
CC	Secretory granule membrane, ficolin-1-rich granule, tertiary granule, specific granule, ficolin-1-rich granule membrane;
MF	Tuberculosis, osteoclast differentiation, toxoplamosis, starch and sucrosemetabolism, galactose metabolism;
KEGG	Acylgycerol O-acyltransferase activity, lysophospholipid acyltransferase, lysophsphatidic acid acyltransferase activity,1-acylglycerol-3-phosphate 0-acyltransferase activity, glucose activity;

### Construct 668 Genes of Blue Module Into Protein-Protein Interaction Network and Analyze Its Biological Function

**FIGURE 5:**
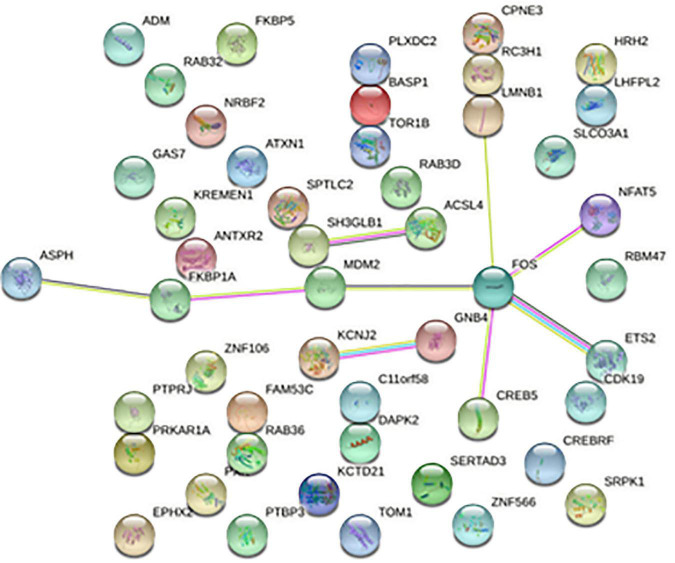
Protein-protein interaction network. Each node represents a protein, and the letter represented is the gene symbol of the corresponding gene. The lines between nodes represent the interaction between two proteins, and the multiple lines between two proteins indicate that there are multiple interaction relationships between the two proteins.

The 668 disease-related genes were used to construct PPI of 564 genes and 104 edge genes in the STRING database and were then visualized using Cytoscape software (Figure 5). In the PPI network, the average node degree was 0.36, and the average local clustering coefficient was 0.158. The analysis results showed that genes related to diseases were mainly expressed in neutrophil, phagocyte, granulocyte, mononuclear cell, and so on. The signaling pathways were mainly enriched in STAT5 activation, interleukin (IL)-10 signaling, neutrophil degranulation, IL-4 and IL-13 signaling, oxidative stress-induced senescence, and innate immune system.

### Identification of the Core 8 Modules by Molecular Complex Detection

**FIGURE 6:**
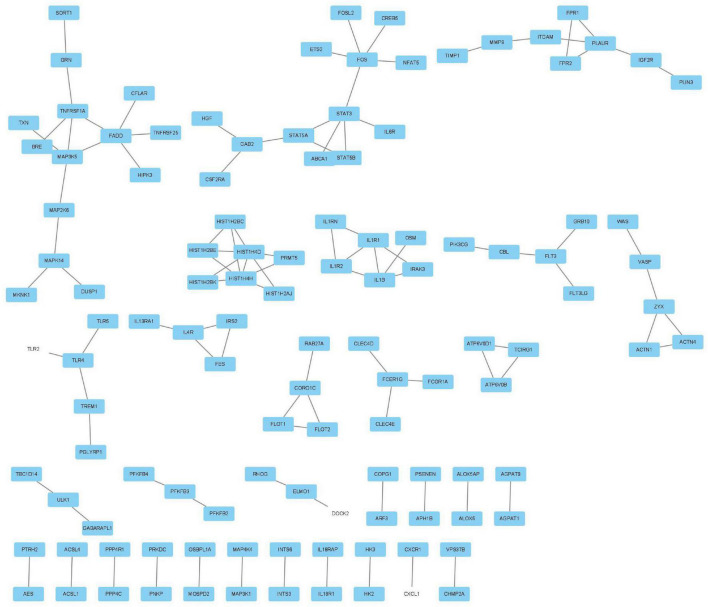
Eight modules, including 21 core genes and 36 marginal genes, were identified by using the molecular complex detection plug-in.

**FIGURE 7:**
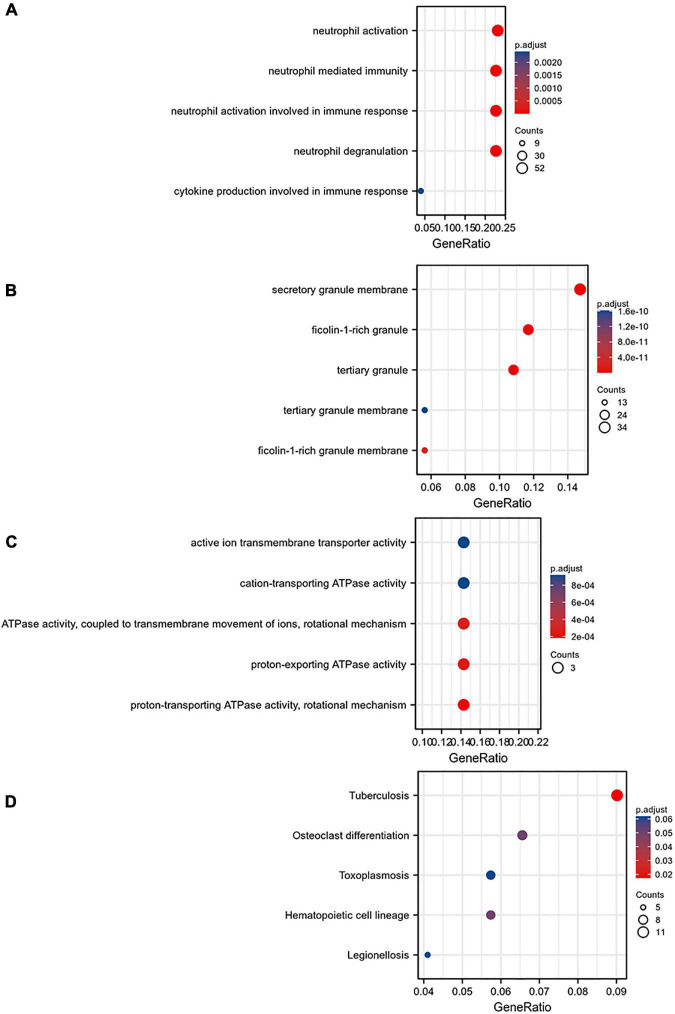
Gene ontology analysis of the eight core modules: **(A)** BP, **(B)** CC, **(C)** MF, and **(D)** KEGG pathway enrichment analysis of the eight core modules.

The MCODE plug-in was used to identify the 8 core modules (Figure 6), including 21 core genes and 36 edge genes, the average node degree was 3.43, and the average local clustering coefficient was 0.692. In addition, the functional analysis of DEGs in the module was performed (*p* < 0.05). In addition, the DEGs in the module were analyzed, and it was found that the core genes and immune functions of the 8 modules have a significant correlation (*p* < 0.05) (Table 2 and Figure 7).

**TABLE 2:** Gene biology function and signal pathway enrichment analysis of eight modules.

GO/KEGG	Function/pathway
BB	Neutrophil activation, neutrophil mediated immunity, neutrophil activation involved in immune response, neutrophil degranulation, cytokine production involved in immune response;
CC	Secretory granule membrane, tertiary granule, specific, ficolin-1-rich granule, tertiary granule membrane;
MF	Active ion transmembrane transporter activity, cation-transporting TAPase activity, ATPase activity, coupled to transmembrane movement of ions, rotation mechanism, proton-exporting ATPase activity, proton-transporting ATPase activity, rotational mechanism;
KEGG	Tuberculosis, osteoclast differentiation, toxoplamosis, Hematopoietic cell lineage, Legionellosis

### Use Venn Diagram to Find the Intersection of Targeted Genes and Disease Genes

**FIGURE 8:**
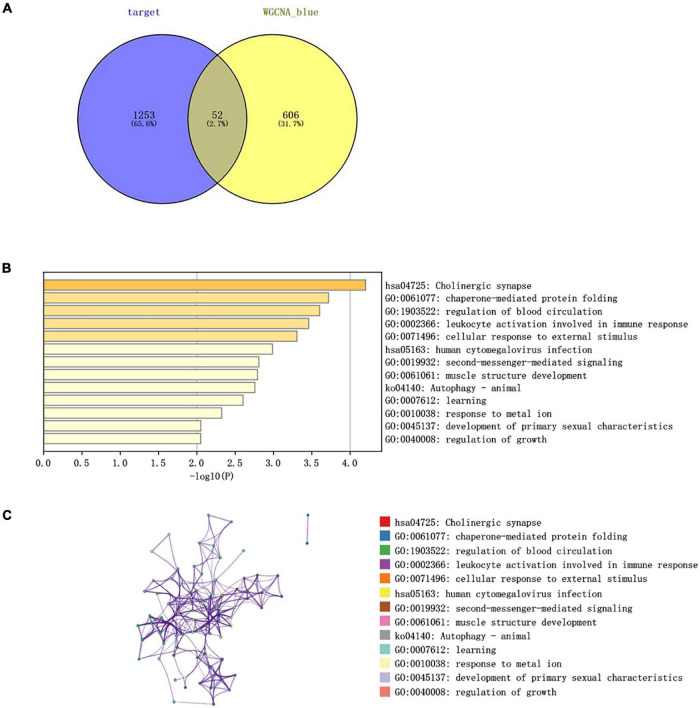
**(A)** Venn diagram constructed based on the blue module gene and the target gene. A total of 52 overlapping genes were identified. **(B)** Heatmap of the enrichment pathways. **(C)** Interaction relationship between the enrichment pathways.

The Venn diagram analysis was used to extract 52 intersecting genes related to disease genes and target mRNAs (Figure 8A). Metascape database^6^ was used for annotation and visualization. Notably, 52 genes were analyzed for GO and KEGG pathway analysis. Min overlap >3 and *p* < 0.01 were considered statistically significant. The GO enrichment results showed that the differential genes were mainly enriched in chaperone-mediated protein folding, regulation of blood circulation, and leukocyte activation involved in immune response; the KEGG enrichment results showed that the differential genes were mainly enriched in the cholinergic synapse, human cytomegalovirus infection, and autophagy-animal pathways. (Figures 8B,C).

### Correlation Between Hub Gene and Immune Infiltration

**FIGURE 9:**
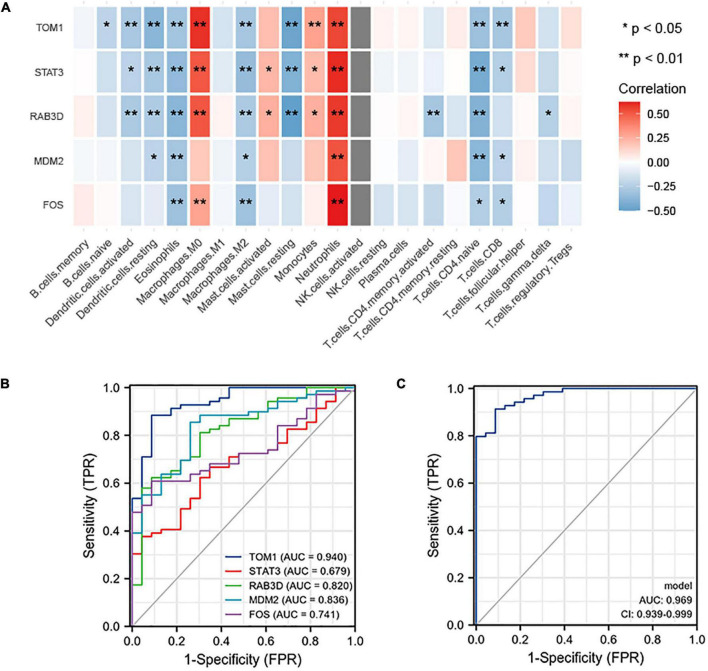
**(A)** Correlation between hub genes and immune infiltration. The top five genes (i.e., TOM1, STAT3, RAB3D, MDM2, and FOS) are all positively related to neutrophils and negatively related to eosinophils. **(B)** ROC prediction sensitivity of 5 core genes (TOM1 showed the highest sensitivity, followed by STAT3, RAB3D, MDM2, and FOS). **(C)** Sensitivity of joint prediction of 5 core genes (area under the curve = 0.969).

NetworkAnalyzer in the Cytoscape (version 3.7.1) was used to analyze the topological parameters in the network. The best target was chosen based on the degree. The top 5 genes were selected as hub genes, namely, TOM1, STAT3, RAB3D, MDM2, and FOS. The correlation between the top 5 genes and the level of immune infiltration was studied. The results showed that hub genes are related to most of the immune cells. For example, the 5 hub genes are significantly positively correlated with neutrophils and significantly negatively correlated with eosinophils (Figure 9A).

### Receiver Operating Characteristic Curve of Hub Gene to Verify Prediction Performance

To understand the diagnostic value of the five core genes, ROC curve analysis was used to calculate the AUC of the gene expression levels in normal control groups and disease groups. In predicting the outcome of the normal control group and stroke, TOM1 has a higher accuracy (AUC = 0.940, CI = 0.887–0.992) in prediction, STAT3 has a lower accuracy (AUC = 0.679, CI = 0.565–0.793) in prediction, RAB3D has a certain accuracy (AUC = 0.820, CI = 0.723–0.916) in prediction, MDM2 has a certain accuracy (AUC = 0.836, CI = 0.747–0.924) in prediction, and FOS has a certain accuracy (AUC = 0.741, CI = 0.641–0.841) in prediction (Figure 9B). The abovementioned 5 hub genes were combined to obtain the ROC curve. In predicting the outcome of control and stroke, the predictive ability of the combined model has a higher accuracy (AUC = 0.969, CI = 0.939–0.999) (Figure 9C).

### Construction of a CircRNA-Mediated ceRNA Regulatory Network in Acute Ischemic Stroke

The CircInteractome online database was used to predict the miRNA targets of statistically different circRNAs. The results showed that there were 213 circRNA-related targeted miRNAs and a total of 405 circRNA-miRNA relationship pairs. By using TargetScan, miRDB, and miRTarBase databases to predict miRNA-related targeted mRNAs, 1,688 miRNA-mRNA relationship pairs were screened.

**FIGURE 10:**
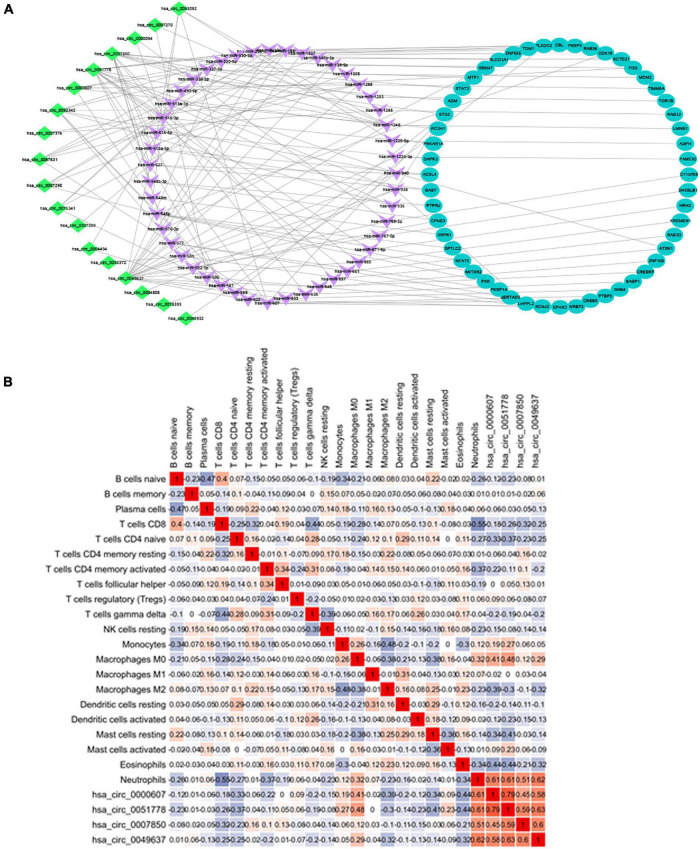
**(A)** ceRNA construction, including 18 circRNA, 47 hsa-miRNA, and 52 mRNA. ceRNA core gene was analyzed using the Metascape database for GO and KEGG enrichment analysis. **(B)** Correlation between immune cells in patients with IS. There is a strong positive correlation between CD8^+^ T cells and initial B cells (0.43), and a strong negative correlation between CD8^+^ T cells and neutrophils (–0.6).

Combining the 52 genes in the Venn diagram, and taking the related circRNA and miRNA at the same time, a total of 132 circRNA-miRNA-mRNA relationship pairs are included. And through Cytoscape software analysis of core genes in circRNA and miRNA competition endogenous network, and successfully constructed circRNA-mediated ceRNA competition endogenous network diagram (Figure 10A).

### The Correlation Between CircRNA and Immune Infiltration

Heatmap was constructed to show the correlation between ceRNA and immune infiltration (Figure 10B). The Pearson’s correlation coefficient was used to analyze the correlation between the key factors in ceRNA and immune cells (non-normal distribution was analyzed by the Spearman’s method), CD8^+^ T cells and hsa-miR-1305 (*r* = −0.310, *p* < 0.005), resting mast cells and hsa-miR-296-5p (*r* = −0.47, *p* < 0.005), macrophages M0 and hsa-miR-296-5p (*r* = 0.59, *p* < 0.005), and neutrophils and hsa-miR-139-5p (*r* = 0.62, *p* < 0.005).

### Use the GSE22255 Dataset to Verify the Hub Gene

**FIGURE 11:**
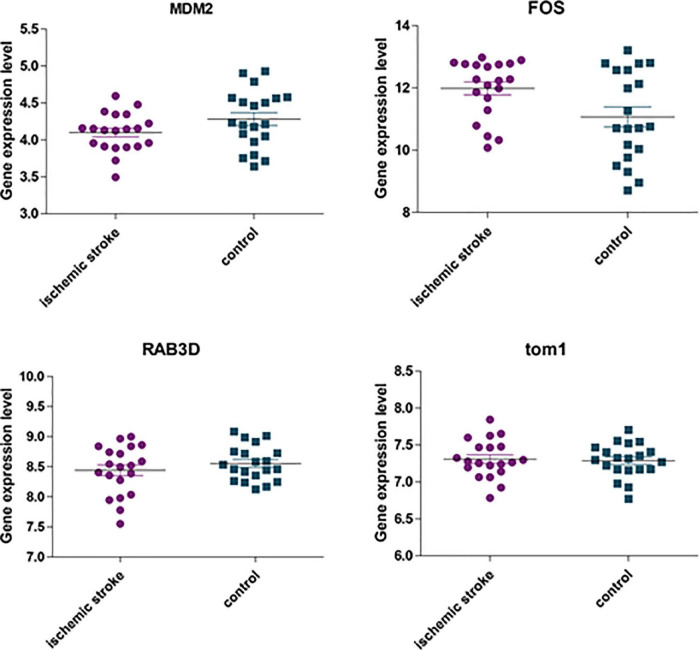
The expression of four hub genes was different between the AIS group and the healthy control group. Among them, *p* = 0.092 in the MDM2 group; *p* = 0.022 in the FOS group; *p* = 0.764 in the TOM1 group; *p* = 0.32 in the RAB3D group.

The GSE22255 dataset was downloaded from the GEO database for verification purposes. The dataset was composed of 20 cases of AIS and 20 healthy controls. The age and gender of the two groups were matched. The 5 genes (i.e., TOM1, STAT3, RAB3D, MDM2, and FOS) were retrieved in the GSE22255 dataset. It was found that there were only 4 genes, i.e., TOM1, MDM2, RAB3D, and FOS. Then, the data were downloaded and analyzed by *t*-test. The result (Figure 11) suggested that FOS genes were upregulated in the AIS group, and there were differences in expression between the AIS group and the control group.

## Discussion

After AIS, neurons in the damaged area release DAMPs, which can trigger the immune response of the infarcted part and recruit/activate glial cells and peripheral white blood cells to the damaged brain area. This can further secrete a variety of pro-inflammatory cytokines, chemokines, and MMPs, resulting in the damage of the blood-brain barrier, and can cause brain edema, hemorrhage transformation, and neuronal necrosis (Rajkovic et al., 2018; Yang et al., 2019). During this process, different immune cells play various regulatory functions at different time points, which affect the prognosis of stroke.

Immune cell infiltration is related to the treatment and clinical outcome of various types of cancer. Evaluating the immune infiltration and determining the differences in immune infiltration components are crucial for the development of new immunotherapeutic drugs that target these cells. This has inspired us to think whether there is a correlation between immune cell infiltration and AIS pathogenesis and treatment. This study found that the immune infiltration levels of naive B cells, naive CD8^+^ T cells, naive CD4^+^ T cells, and resting mast cells were downregulated in patients with AIS, while the levels of CD4 memory-activated T cells, macrophages M0, and neutrophils immune infiltration were upregulated. Notably, the changes of CD8^+^ T-cell level were strongly positively correlated with naive B cells and were in a synergistic relationship. Resting NK cells were moderately positively correlated with memory B cells. Memory CD4^+^ T cells were moderately positively correlated with gamma-delta T cells. Naive B cells were correlated with resting dendritic cells and showed a moderately positive correlation change, where their possible mechanism of relationships is rarely reported. We infer that the changes of CD8^+^ T cells and the naïve B cells enhance each other’s effects during stroke, where further study on the correction of these immune cells is needed.

In the early stage of AIS, the activated M1 macrophages secrete a large amount of pro-inflammatory cytokines to promote the adhesion and exudation of neutrophils, lymphocytes, and monocytes. In the late stage of AIS, the replacement of activated M2 macrophages plays a major role, which secretes protective remodeling factors and promotes axon growth and angiogenesis (Ritzel et al., 2018); 24 h after AIS, the specific immune response in the body is activated (Haeusler et al., 2012), when the course of the disease enters the advanced stage. Due to the increased permeability of the blood-brain barrier in the early stage of stroke, lymphocytes enter the central nervous system after stroke and induce autoimmune reactions by recognizing antigens in the central nervous system. Studies have shown that the levels of CD4^+^, CD8^+^ T cells, and CD4^+^/CD8^+^ in the peripheral blood of patients with stroke are closely related to the severity and prognosis of AIS (Dolati et al., 2018). CD4^+^ T cells secrete cytokines, such as interferon (IFN)-γ and IL-17, in a non-antigen-dependent manner and promote the expression of chemokines by glial cells in the brain, enhancing neutrophils infiltration and aggravating the early nerve damage of AIS. In addition, T lymphocytes can mediate neurocytotoxicity. Regulatory T cells can be detected in large quantities in infarct tissues on 7 and 14 days after cerebral ischemia. The mechanism is that regulatory T cells release immunosuppression factors that regulate brain damage and maintain the body’s immune homeostasis, which forms the foundation for the reconstruction of the structure and function of the later stage of brain injury parts (Zhang et al., 2020; Shi et al., 2021). Animal studies found that regulatory T cells infiltrated the brain of mice 1–5 weeks after stroke. The selective depletion of regulatory T cells reduces oligodendrocyte production, white matter repair, and functional recovery after stroke. Increasing the levels of IL-2 and IL-2 antibody complexes after stroke can increase the number of regulatory T cells, which can improve white matter integrity in the long term and protect nerve functions (Li et al., 2020).

In addition, B lymphocytes are also involved in the immune response after stroke, but the mechanism is not clear. It is proposed that B lymphocytes secrete IL-10, which reduces the entry of activated inflammatory T cells into the ischemic brain tissue and increases CD8^+^CD122^+^Treg cells, and play a role in nerve protection (Stamova et al., 2014). After the stroke, the number of immune cells in the ischemic brain tissue and peripheral organs changes dynamically, and the expression levels of cell surface markers indicate that the brain is at the post-stroke inflammatory response state.

CircRNA-regulated ceRNA is a circRNA-miRNA-mRNA regulatory network constructed based on the regulatory and regulated relationship between different RNAs (Qu et al., 2015), and its possible mechanism is that there are a variety of miRNA response elements (MREs). miRNA binds to targeted mRNA through MRE and then plays a regulatory role in mRNA by inhibiting the translation of mRNA or reducing mRNA. Studies have shown that MRE also exists on circRNA. When circRNA and mRNA have the same MRE, the two will compete for the same type of miRNA after the disease occurs (Bosson et al., 2014). Another important biological function of circRNA is to act as the sponge of miRNA and indirectly regulate gene expression (Salmena et al., 2011). As a new form of post-transcriptional regulation, ceRNA has a great potential in disease research. Several studies have shown that the ceRNA regulatory network may be associated with cancer, atherosclerosis, heart disease, diabetes, and other diseases (Chan and Tay, 2018; Yan et al., 2020; Dai and Zhong, 2021; Liu et al., 2021), especially in AIS disease, where it plays an important regulatory role.

In our study, 559 differential circRNAs were identified in the AIS group and the normal group, including 213 upregulated circRNA and 346 downregulated circRNA. A total of 668 core genes were obtained from the GSE58294 dataset, as described previously. Three different methods were then used to find and verify the core genes and study their biological functions (1) using direct GO and KEGG analysis, (2) constructing a PPI network, and (3) using the MCODE plug-in to identify the 8 core modules. Its main biological functions are enriched in the activation of neutrophils, immune regulation of neutrophils, and other inflammatory factors. Most of the biological pathways involved are rich in immune and inflammatory response-related pathways, including primary immune-related pathways and immune diseases, and cytokine activation-related pathways. After multiple verifications of the target gene in this study, it is found that its biological function and signal pathway enrichment are mostly related to immune-inflammatory factors. It is speculated that the target gene plays an important function in the immune response mechanism of AIS.

The Venn diagram analysis was used to identify the disease gene and target mRNA-related intersection genes (a total of 52), and GO and KEGG pathway analysis were used to find that they are related to leukocyte activation involved in immune response and autophagy-animal pathway.

After AIS, the white blood cell count increases significantly (Emsley et al., 2003), and it is closely related to the patient’s poor prognosis (Furlan et al., 2014). As the main subtype of white blood cells, neutrophils accumulate in the cerebral blood vessels within a few hours under the action of attracting chemokines, such as CCL5, cxcl1, cxcl2, and cxcl3 (Cai et al., 2020; Kang et al., 2020), which may cause the expansion of the infarct and block the capillaries (McColl et al., 2008). In contrast, neutrophils regulate neuroinflammation after stroke through various inflammatory mediators, such as neutrophil protease (NE), myeloperoxidase (MPO), MMP, tissue inhibitors of metalloproteinase (TIMP), IFN, and IL (Cai et al., 2020), especially the expression of MMP can directly destroy the blood-brain barrier, leading to secondary brain injury or hemorrhagic transformation (McColl et al., 2008). Another feature of neutrophils is the formation of neutrophil extracellular traps (NETs). Studies have confirmed that the presence of NETs is a predictor of poor outcomes in myocardial infarction (Mangold et al., 2021), and inhibiting the formation of NETs can effectively prevent myocardial ischemia (Vajen et al., 2018). When a stroke occurs, neutrophils accumulate in the ischemic area and release toxic signals, such as NETs, thereby inhibiting revascularization and vascular repair after stroke (Kang et al., 2020). Studies have found that when patients with acute cerebral infarction are admitted to the hospital, their severity is related to a higher total number of white blood cells and neutrophil counts (Tarkanyi et al., 2020).

This study further analyzed the correlation between the intersection genes and the level of immune infiltration. The top 5 hub genes were TOM1, STAT3, RAB3D, MDM2, and FOS, and they were all positively correlated with neutrophils and negatively correlated with eosinophils. FOS, Rab3D (Nishio et al., 1999), and MDM-2 (Wang H. et al., 2021) genes have been reported to be correlated with neutrophils. There are few studies on the relationship between eosinophils and stroke, but there is evidence that the eosinophil count is valuable for predicting the recurrence and prognosis of cerebral infarction, and its decrease is associated with the AIS severity and clinical outcome at 3 months (Wang et al., 2017; Zhao et al., 2019), although the specific mechanism is unclear. The author speculates that eosinophils may be related to stroke through these 5 hub genes, but further research is needed. To verify the sensitivity of the 5 hub genes to disease diagnosis, ROC curve diagnostic test shows that the 5 hub genes have good performance in disease diagnosis, and the joint prediction of 5 hub genes has a higher sensitivity and a positive prediction rate. It also provides theoretical support for clinical diagnosis.

We further used the GSE22255 dataset to verify the 5 hub genes. Among them, STAT3 was not found. Among the other genes, only FOS was statistically different between the AIS group and the control group. It may be related to the dataset we selected. The GSE58294 dataset has many cases and is representative, but AIS is a cardiogenic stroke. Other datasets do not specify which type of stroke it is, which will lead to certain limitations in the conclusion. However, from the abovementioned analysis results, we speculate that whether it is cardiogenic AIS or other types of stroke, FOS is a gene worthy of attention and may play a regulatory role in the immune infiltration mechanism in IS.

By constructing a circRNA-mediated ceRNA regulatory network, 18 circRNAs related to AIS immune mechanisms were identified, among which hsa-circ-0000607, hsa-circ-0051778, hsa-circ-0007850, and hsa-circ-0049637 showed the strongest correlation with neutrophils. At present, there are few related studies on these four circRNAs, and the relationship with immunity has not been reported. However, through the ceRNA regulatory network, we found that hsa-circ-0049637 and FOS gene are correlated, and hsa-circ-0000607, hsa-circ-0051778, hsa-circ-0007850, and LMNB1 are correlated.

The FOS protein is a cell transcriptional regulatory factor. After the cell is stimulated, the c-fos gene in the nucleus is activated, is transcribed into mRNA, enters the cytoplasm, and is translated and synthesized into nucleophosphate protein. As an immediate early gene, c-fos is activated instantaneously and rapidly in response to a variety of cell stimuli. In different rat models, it can reach its peak level in 2–4 h after cerebral ischemia. The C-fos level can then gradually return to normal levels within 24 h (Karch et al., 2013; Nikoletopoulou et al., 2013). Studies have shown that its expression is parallel to the death or apoptosis of neurons after cerebral ischemia, that is, the degree of expression reflects the degree of neuronal death or apoptosis after stroke. The higher the c-fos expression level, the more serious the corresponding brain tissue damage would be (Whitfield et al., 2001). The main function of FOS is that it forms heterodimer FOS-jun complex with jun nuclear protein, which becomes the main form of transcriptional activator protein-1 (AP-1) when the cell is stimulated. The latter is in the AP-1-specific sites of the promoter and enhancer regions. It can regulate gene expression by binding to DNA and transforming extracellular signals into the nucleus (Bejjani et al., 2019). While neutrophils can be recruited from blood vessels to the site of inflammation, chemokines play an important role. In acute lung injury (ALI), the p38MAPK signaling pathway can regulate various inflammatory response processes. AP-1 is a downstream transcription factor of p38MAPK. The activated p38MAPK further activates AP-1 in the nucleus to regulate the release of downstream inflammatory mediators (e.g., chemokines, IL-1β, tumor necrosis factor-α, and adhesion molecules), which then activate many neutrophils, causing them to infiltrate at the inflammation site, and damage alveolar epithelial cells and lung capillary endothelial cells (Kumari et al., 2015).

LMNB1 is a nuclear lamin, which is closely related to the nuclear membrane, chromatin, nuclear pore complex, and nuclear matrix in the structure. It not only provides a structural scaffolding for nuclear membrane and chromatin but also plays a role in DNA replication, mRNA sorting, and cell mitosis. During the process, the nuclear membrane’s disintegration and reformation play an important role (Gruenbaum et al., 2005; Lees-Miller, 2006). Current studies have shown that Lmnb1, as a substrate for autophagy in primary human cells, can interact with SIRT1 (Wang L. et al., 2021) and autophagy protein LC3/Atg8 (Dou et al., 2015), which plays a regulatory function in human aging. When using the Metascape database for GO and KEGG enrichment analysis of 52 target genes, it was found that one of the pathways was enriched in the autophagy-animal signaling pathway. Studies have shown that in pMCAO rats, the use of glycogen synthase inhibitor-3β (GSK-3β) can reduce the inflammatory response caused by ischemic injury and detect a higher level of autophagy. In cells cultured *in vitro*, the use of autophagy inhibitor Beclin-1 siRNA can increase the inflammatory response of microglia. Studies have shown that GSK-3β can inhibit neuroinflammation induced by ischemic brain injury in rats by activating autophagy (Zhou et al., 2011). In the MCAO/R model, GSK-3β can increase the activity of autophagy by inhibiting the inflammatory protein NLRP3, thereby reducing ischemia-reperfusion injury (Wang et al., 2019). Many other studies (Cho et al., 2014; Yang et al., 2015; Hong et al., 2018) have proved that in IS, autophagy is closely related to the inflammatory response (Green et al., 2011), and this correlation may provide a new direction for the treatment of IS. Although the four circRNAs have not been reported to be related to inflammation, the corresponding proteins have been previously reported to be related to inflammationOur method also has some limitations. Due to the inconsistent circRNA naming, some circRNA will be lost during the data integration process, which may reduce our results. Another limitation is that due to the small number of samples from AIS patients, some of the identified circRNAs may be false positives, and the choice of the dataset may cause the results to have certain limitations. In future studies, we will use more samples to confirm the role of circRNA in the immune mechanism of AIS.

In summary, after patients with AIS, the initial B cells, initial CD8^+^ T cells, initial CD4^+^ T cells, and resting mast cell immune infiltration levels were downregulated, while CD4 memory activated T cells, macrophages M0, and neutrophils immune infiltration levels were increased. The hub genes related to the level of immune cell infiltration are TOM1, STAT3, RAB3D, MDM2, and FOS, and they have a significant positive correlation with neutrophils and a negative correlation with eosinophils. By constructing a circRNA-mediated ceRNA regulatory network, 18 circRNAs related to the immune mechanism of AIS were identified, of which four circRNAs, namely, hsa-circ-0000607, hsa-circ-0051778, hsa-circ-0007850, and hsa-circ-0049637, have the strongest positive correlation with neutrophils. The distribution characteristics of immune infiltration in patients with AIS discovered in this study and the identified circRNA may provide new research ideas for IS treatment, prevention, and new drug development.

## Data Availability Statement

The original contributions presented in the study are included in the article/supplementary material, further inquiries can be directed to the corresponding author/s.

## Author Contributions

ZM contributed to the data analysis, manuscript writing, and submission. C-FL, LZ, NX, and YZ contributed to the review and revision of the manuscript. LC contributed to the study design. All authors contributed to the article and approved the submitted version.

## Conflict of Interest

The authors declare that the research was conducted in the absence of any commercial or financial relationships that could be construed as a potential conflict of interest.

## Publisher’s Note

All claims expressed in this article are solely those of the authors and do not necessarily represent those of their affiliated organizations, or those of the publisher, the editors and the reviewers. Any product that may be evaluated in this article, or claim that may be made by its manufacturer, is not guaranteed or endorsed by the publisher.

## Footnotes

1
http://cibersort.stanford.edu/

2
https://www.ncbi.nlm.nih.gov/geo/

3
http://david.ncifcrf.gov/

4
http://string-db.org

5
https://circinteractome.irp.nia.nih.gov/

6
www.metascape.org
